# Book Review

**Published:** 1896-06

**Authors:** 


**A Few Medical and Surgical Reminiscences.** By AUGUSTIN PRICHARD. Pp. 99. Bristol: J. W. Arrowsmith. 1896.—The writer of this little book is endowed with a vivid and retentive memory, which has enabled him to put on record some delightful "reminiscences." A graphic description of his early youth and education is one of the most interesting parts of his book. Mr. Prichard had the good fortune to be well-born and well-bred on both sides for his intended profession. Medicine was represented by his father, the celebrated Dr. James Cowles Prichard, whose work, *The Researches into the Physical History of Man*, gained him an European reputation and the Fellowship of the Royal Society. Surgery was represented by his maternal uncle, Mr. John Bishop Estlin, the founder of the Bristol Eye Dispensary, and to whom Mr. Prichard, while still at school at the Bristol College, was apprenticed in 1834. In 1836 he entered the Bristol Royal Infirmary as a medical pupil under his father, and soon afterwards as a surgical pupil under Mr. Harrison. Mr. Prichard gives an entertaining description of the effect which, in his pupil days, the theories of Broussais had on the treatment of inflammatory diseases, resulting in the innumerable bleedings, cuppings, and leechings which formed the regular routine of infirmary practice at that time, but which remedies, especially when applied locally, have in the present day fallen too much into undeserved oblivion. Dr. Prichard being of such general culture and well versed in foreign languages, would be fully alive to the advantage of Continental travel for his son, and accordingly Mr. Prichard, after attending at St. Bartholomew's, continued his medical education at Berlin (where he took the M.D.), Vienna, and Paris. We get very interesting reminiscences not only of many of the great physicians and surgeons of London during the time Mr. Prichard was a student there, but also of Dieffenbach, Müller, Rokitansky, Jäger, Cruveilhier, Louis, Civiale, Velpeau, and many others. In 1842 he returned to Bristol and began practice. Mr. Prichard's pictures of medical men and manners fifty years ago are admirable portraits, full of life and character; and all his descriptions are pervaded with a quiet humour which renders the book exceedingly readable. An excellent photogravure portrait of the writer forms the frontispiece to his volume.

**The Medical Digest.** Appendix, including the years 1891–2–3–4, and to August, 1895. By RICHARD NEALE, M.D. London: Ledger, Smith and Co. 1895.—Neale's *Medical Digest* is too well known to require a long notice. This supplementary volume is a welcome one, and we heartily recommend to every member of the profession this "busy practitioner's *vade-mecum*." Although to a large extent superseded by fuller and more recent bibliographies, its arrangements are so simple that little time is necessary to find a required reference in the periodicals with which it deals. This volume, like its predecessors, is provided with an excellent index. It has two pages of *corrigenda* of the 1890 edition.

**Elements of Practical Medicine.** By ALFRED H. CARTER, M.D. Seventh Edition. Pp. xvi., 552. London: H. K. Lewis. 1895.—That this concentrated extract of medicine is clearly appreciated is shown by the fact that in fifteen years six large editions have been sold. As a simple introduction to the study of medicine it quite fulfils the aim of its author, and he who has digested it well cannot fail to have acquired a taste for more.

**An Essay on Malaria and its Consequences.** By ROBERT LINDSAY, M.B. Pp. 116. London: H. K. Lewis. 1895.—This monograph by Dr. Lindsay, a retired surgeon of the

Army Medical Department, is an attempt to show that all our preconceived ideas about malarial fevers are entirely in the wrong. The book takes no notice of the work which has lately been done by Laveran and others. It ignores entirely the discovery of the malaria bacillus in the blood, and attributes the whole of the morbid phenomena of malaria to an excess of carbonic acid in the atmosphere. The author draws an analogy between the symptoms of acute poisoning by carbonic acid gas and those observed in malarial poisoning, both acute and chronic, and founds upon this his theory that malarial fevers and all their complicated sequelæ are neither more nor less than the expression of the effect of continuous poisoning of the blood by carbonic acid. This is the theory, very feebly sustained. It is needless to say the deductions from it are equally feeble. The treatment of malarial fevers is to be radically altered; quinine is to be given up; the bromides and the chlorides are the author's anchors of hope. It is useless to say more. It is a pity such books are written.

**On Dreamy Mental States.** By Sir JAMES CRICHTON-BROWNE, M.D., F.R.S. Pp. 32. London: Baillière, Tindall and Cox. 1895.—In this, the Cavendish Lecture delivered before the West London Medico-Chirurgical Society, we have a consideration of some of those indefinite mental conditions which are on the borderland of pathology, and some of which are very common, although not often described. One is, in Dickens's words, the "feeling which comes over us occasionally of what we are saying or doing having been done in a remote time, of our knowing perfectly well what will be said next, as if we suddenly remembered it." Another is the curious impression of loss of personal identity which has been described by various writers, *e.g.* Tennyson and John Addington Symonds. These states involve, we are told, "disorder of mind, trifling and transient no doubt, like cramp of a few fibres of a muscle, but disorder nevertheless;" and they may be looked upon, the lecturer informs us, as allied to the milder forms of *petit mal*. Connected with this are the feelings experienced just before chloroform and nitrous oxide anæsthesia, which consist frequently in "convictions of emancipation, relief, and happiness, in grand and sublime ideas." These and kindred conditions are described and discussed in a most interesting manner, and with a command of language which makes the most intricate psychological phenomena as clear as such things can be made, for they baffle accurate analysis. "Plunges they are into those depths of outer mystery in which the certitudes of Science lose themselves, and out of which, it has been said, the certitudes of faith arise."

**On Appendicitis, and on Perforation of Gastric and Duodenal Ulcer.** By GILBERT BARLING, M.B., B.S. Pp. 92. Birmingham: Cornish Brothers. 1895.—This book consists of the 1895

Ingleby Lectures, reprinted from the *Birmingham Medical Review*. It contains much that is good, though not much that is new. The author, in common with most British surgeons, takes an eminently conservative view of the treatment of appendicitis, and is by no means disposed to advise the radical methods advocated on the other side of the Atlantic. The recommendations are sound and are marked by much good sense, and the practical surgeon may find many useful hints by a careful perusal of these lectures. The author should have provided his reprint with an index.

**Observations on Congenital Dislocation of the Hip.** By Bernard E. Brodhurst. Second Edition. Pp. 19. London: J. & A. Churchill. 1895.—The author says that fifty-two cases of this form of dislocation have come under his care during the last thirty-five years. He maintains that they are for the most part traumatic, and are either produced *in utero* or more commonly at birth. He advocates operation whenever an acetabulum can be made out by an exploring needle. The essay is a very good summary of what is known of this curious affection.

**Methods of Operating for Cataract.** By Surgeon-Captain G. H. Fink. Pp. 77. London: J. & A. Churchill. 1894.—This little book is based upon the results of some five hundred operations. Iridectomy, as part of the operation, is recommended with hard well-matured cataracts, not with cortical and fluid cataracts. The author advises it if there is any suggestion of inflammation in the cornea, or any tendency towards glaucoma. Where everything is favourable he advocates no iridectomy. He applies a plaster to tightly seal the lids together, allowing drainage from the conjunctival sac by means of slits through the plaster opposite the canthus. The plaster is left for four days; it is then replaced by a pad and bandage, and at a week after the operation by a shade only. Our author does not hesitate to extract cataract from both eyes at one sitting; but the occurrence of 6 per cent. cases of suppuration is a large proportion, particularly as he is an enthusiastic disciple of Listerian principles.

**Regulations as to Defects of Vision and other Physical Defects which Disqualify Candidates for Admission into the Civil or Military Government Services.** By N. C. Macnamara. Pp. 31. London: J. & A. Churchill. 1895.—This valuable monograph gives good rules for deciding whether a boy is likely to fail by physical imperfections from qualifying for some selected branch of the Government service. The regulations regarding eyesight are thoroughly traversed. Points to be observed regarding physical defects and the method in which physical examination is conducted are well discussed and explained. Such a manual should be consulted by all who propose to enter a boy for the public services before the school curriculum is modified, for a physical standard is necessary as well as an intellectual one.

**Notes on Surgery for Nurses.** By Joseph Bell, M.D. Fourth Edition. Pp. 180. Edinburgh: Oliver and Boyd. 1895.—The fact that this little work has reached a fourth edition proves that it has been appreciated, and we do not doubt that it will continue a favourite companion of nurses and probationers. A chapter has now been added entitled "General Advice to Nurses," which contains much good sound common-sense, and which will render the book still more useful to those for whom it is designed.

**Guy's Hospital Reports.** Vol. LI. London: J. & A. Churchill. 1895.—The present volume is a smaller one than usual, and is scarcely equal in interest to some of its predecessors. Mr. Thomas Bryant contributes a short paper on "Temperature after Operations," showing that he had obtained very good results after most of his operations without the use of the carbolic spray, but with the use of iodine water for washing the wounds. Dr. Theodore Fisher contributes an important paper on "Hypertrophy of the Heart without Gross Organic Lesion," in which he maintains that overwork and addiction to alcohol are frequently the causes of considerable hypertrophy of the heart without any valvular disease or other organic lesion. The paper is illustrated by numerous clinical and pathological records from the Bristol General Hospital and from Guy's Hospital. "A Case of Multiple Atrophying Sarcomata of the Head and Neck," by L. A. Dunn, is interesting, and so are papers by F. Newland-Pedley on "The Treatment of Suppuration of the Maxillary Antrum" and "On the Treatment of Congenital Cleft Palate."

**King's College Hospital Reports.** Vol. I. London: Adlard and Son. 1895.—We congratulate the editors of this new set of hospital reports on the general excellence of the first volume. It contains several interesting original papers and the reports of each department of the hospital, with full accounts of some of the most valuable cases. The volume will be of special interest to old King's College men, but it is well worthy of perusal as a record of work fully up-to-date in all particulars.

**Medico-Chirurgical Transactions.** Vol. LXXVIII. London: Longmans, Green and Co. 1895.—This volume contains no less than fourteen monographs on a variety of topics, of which perhaps the most noteworthy is that on the influence of heredity in phthisis, by Dr. J. Edward Squire, who states that the analysis of 1000 cases warrants the opinion "that the hereditary predisposition in the case of phthisis is not a direct predisposition to this malady, but merely the general predisposition to disease which belongs to the offspring of weakly parents, a predisposition which the children of phthisical parents have in common with those of parents whose health is lowered by other conditions. The families of consumptives have, however, the disadvantage, not shared by the delicate children of non-phthisical parents, of living in constant association with a source of infection."

**Transactions of the Grant College Medical Society, Bombay.** From January to December, 1894. Bombay: The "Tatva-Vivechaka" Press. 1895.—We are glad to receive this record of the activity and energy of the 168 members—for the most part Indian—of the Grant College Medical Society at Bombay. It contains the report of a lengthy debate on Pasteurism and Bacteriology. If there should be another volume in the future, it should have a table of contents and an index.

**Intercolonial Medical Journal of Australasia.** Melbourne: Stillwell & Co.—This new monthly, beginning its issue with the present year, results from the amalgamation of the *Australian Medical Journal* and the *Intercolonial Quarterly Journal of Medicine and Surgery*. It is intended that the new journal shall be truly federal in character and absolutely free from the taint of localism. The medical profession throughout Australasia are invited to become contributors and subscribers to a journal which has an excellent editorial staff, and which should have the confidence of a very wide constituency.

'Ιατρική 'Επιθεώρησις. Athens: 40 Rue Zinonos.—We offer a word of warm welcome to a recent addition to the ranks of cosmopolitan medical literature. It has been strongly urged that modern Greek should be employed as the medium of universal scientific intercommunication—a suggestion which seems highly reasonable after the perusal of some numbers of this weekly journal, the first number of which appeared at the beginning of the year. A paragraph in the issue for January 31st, 1896, headed "'Η *ἀνακάλυψις τοῦ* Röntgen.—'Η *διὰ μέσου στερεῶν σωμάτων φωτογράφησις*" suffices to show how the language of Hippocrates and Aristotle has been expanded to meet the requirements of modern science and medicine. Our contemporary does not present much material originating in Athens; neither Greek Society nor meeting is reported in its columns, and the Medical School and Children's Hospital are the only institutions we see mentioned. The two Professors who superintend the publication supply the only original work, the rest being made up mainly of reports of Parisian societies and extracts from other journals. This, as well as the matter of printing and correcting for the press, no doubt will improve with time. We wish *Medical Observation* all success!

**The Medical Annual.** Bristol: John Wright & Co. 1896.—This is an exceedingly good number of this well-known *Annual*. It contains a large and well-selected amount of information respecting new remedies and new methods of treatment, gathered from the writings of numerous authors. It also contains some very useful original articles written by specialists in different departments of medical work. Naturally, these articles vary in value directly with the practical acquaintance with the special work to which they refer; but even where they are produced by abstracting from the published work of men of more experience than their authors, yet this abstracting has been well done. The volume is well illustrated, and some of the illustrations are excellent; and even cycling has a special chapter devoted to it. We thoroughly recommend the book as one that contains a great amount of information, which is given to us in a readable style. It would be an improvement if a uniform method were adopted in quoting the references, and if the nomenclature of the *Index Medicus*, the best existing bibliographical standard, were employed for the titles of the journals.

**Archives of Clinical Skiagraphy.** By SYDNEY ROWLAND. Vol. I., Part I. May, 1896. London: The Rebman Publishing Company, Limited.—It is characteristic of the present generation that an invention or discovery which half a century ago would have required years for its full development is nowadays often matured in as many months. Of this we have recently had a striking example in the "New Photography." Although it is only a few months since Röntgen announced his discovery of the X rays and their property of penetrating more or less completely substances opaque to ordinary light, the application of this property to the photography of the bones and other dense portions of the human body has already passed its first purely experimental stage, and is coming to be a recognised aid to medical diagnosis. Mr. Sydney Rowland, who is well known to readers of the *British Medical Journal* as an able exponent of the new art (named by him Skiagraphy), has just published the first number of the *Archives of Clinical Skiagraphy*. This he describes as "a series of collotype illustrations with descriptive text, illustrating applications of the New Photography to medicine and surgery." It contains excellent skiagrams of the body of a child aged three months (showing that the intestines, heart, and liver cast shadows as well as the bones); of an arm with syphilitic disease of the radius; of a hand with needle in one of the fingers; and of the hand and knee-joint of a girl suffering from multiple exostoses. In connection with the latter, Mr. Rowland points out that the diseased bones are unusually transparent to the X rays. The same thing was noticed in a similar case recently photographed at University College, Bristol. So marked was the effect, that after several attempts had been made a normal knee was photographed for comparison before it was realised that the bone and not the apparatus was the cause of the faintness of the bone-shadows. It is, we think, a pity that Mr. Rowland has painted out the tissues before printing his negative of the knee-joint, as the absence of contrast between them and the bones is thereby missed. The practice of touching up negatives taken for scientific purposes always tends to shake one's faith in their accuracy. The printing and paper of the *Archives* are all that can be desired, and the reproductions of the skiagrams clear and good. A neat portfolio is provided for containing the numbers.

**The Advertiser's A B C.** 1896. Pp. 1162. London: T. B. Browne, Ltd.—Some years ago we had the opportunity of reviewing a previous edition of this magnificent volume, and spoke in high praise, not only of the value it must be to all advertisers, but also of the beautiful manner in which the book has been produced; and in these respects the present issue is no whit behind its predecessors. Its coloured reproductions of magazine covers and reduced facsimiles of newspaper pages are specially attractive. There is an article at the commencement of the book, entitled "Non-Advertising Professions," in which the medical, amongst others, comes in for severe castigation. We cannot here discuss the *pros* and *cons* of advertising by medical men of their powers to cure certain diseases, &c., as the writer wishes them to do, but we can assure him that though it may not be done through what he would call the legitimate channel—*i.e.* the advertising agent,—it is done to no inconsiderable extent by other means, which reflect little credit on the advertisers. The writer's case is not made stronger by his sympathetic references to two notorious quacks whose names have been removed from the *Medical Register* for "infamous conduct in a professional respect," *i.e.* advertising their nostrums and reputed cures in lay papers. We hope we shall not see the day when medical advertising is considered anything else than it is now—an ignoble and debasing act, and one unworthy of a great and noble profession. In a work of this magnitude there must, of course, be errors; but *The Journal of Anatomy and Physiology* ought not to have been named as one of the "new ventures" of 1895. On page 386 a mistake has occurred in the name of our Editorial Secretary.

